# Inflammatory response and parasite regulation in acute toxoplasmosis: the role of P2X7 receptor in controlling virulent atypical genotype strain of *Toxoplasma gondii*

**DOI:** 10.3389/fimmu.2024.1452828

**Published:** 2024-08-29

**Authors:** Thuany Prado-Rangel, Aline Cristina Abreu Moreira-Souza, Sthefani Rodrigues Batista da Silva, Thais Barboza-Araujo, Archimedes Barbosa Castro-Junior, Isalira Peroba Rezende Ramos, Christina Maeda Takiya, Rossiane Claudia Vommaro, Robson Coutinho-Silva

**Affiliations:** ^1^ Laboratório de Imunofisiolofia, Instituto de Biofísica Carlos Chagas Filho, Universidade Federal do Rio de Janeiro, Rio de Janeiro, Brazil; ^2^ Laboratório de Ultraestrutura Celular Hertha Meyer, Instituto de Biofísica Carlos Chagas Filho, Universidade Federal do Rio de Janeiro, Rio de Janeiro, Brazil; ^3^ Department of Biomedical Sciences, Arthur A. Dugoni School of Dentistry, University of the Pacific, San Francisco, CA, United States; ^4^ Centro Nacional de Biologia Estrutural e Bioimagem-CENABIO, Universidade Federal do Rio de Janeiro, UFRJ, Rio de Janeiro, Brazil; ^5^ Laboratório de Imunopatologia, Instituto de Biofísica Carlos Chagas Filho, Universidade Federal do Rio de Janeiro, Rio de Janeiro, Brazil

**Keywords:** toxoplasmosis, Brazilian strain, purinergic signaling, inflammation, EGS strain, parasite control

## Abstract

Toxoplasmosis is a globally significant disease that poses a severe threat to immunocompromised individuals, especially in Brazil, where a high prevalence of virulent and atypical strains of *Toxoplasma gondii* is observed. In 1998, the EGS strain, exhibiting a unique infection phenotype, was isolated in Brazil, adding to the complexity of strain diversity. The P2X7 receptor is critical in inflammation and controlling intracellular microorganisms such as *T. gondii.* However, its genetic variability can result in receptor dysfunction, potentially worsening susceptibility. This study investigates the role of the P2X7 receptor during acute infection induced by the EGS atypical strain, offering insight into the mechanisms of *T. gondii* infection in this context. We infected the female C57BL/6 (WT) or P2X7 knockout (P2X7^−/−^) by gavage. The EGS infection causes intestinal inflammation. The P2X7^−/−^ mice presented higher parasite load in the intestine, spleen, and liver. The absence of the P2X7 receptor disrupts inflammatory cell balance by reducing NLRP3, IL-1β, and Foxp3 expression while increasing IFN-γ expression and production in the intestine. In the liver, P2X7^-/-^ animals demonstrate diminished inflammatory infiltrate within the portal and lobular regions concurrent with an enlargement of the spleen. In conclusion, the infection of mice with the EGS strain elicited immune alterations, leading to acute inflammation and cytokine dysregulation, while the P2X7 receptor conferred protection against parasitic proliferation across multiple organs.

## 1 Introduction

Toxoplasmosis is a significant disease worldwide, which poses a severe concern for immunocompromised patients, such as HIV-positive patients, transplant recipients, and those undergoing cancer treatment, among others (1, 2). Brazil presents a high prevalence of virulent and atypical strains. Clinical manifestations involve severe ocular damage and multiple organ failure, even in immunocompetent patients, and lead to significant congenital damage (3). Outbreaks of toxoplasmosis recur in Brazil, and strains with an atypical genotype profile continue to be isolated from affected patients during these outbreaks (4).

The genetic variants in Brazil were associated with a higher prevalence of eye disease compared to other regions of the world (5). In most Brazilian areas, 50–80% of pregnant women presented seroprevalence for toxoplasmosis, and children’s eye injuries are more frequent than in Europe (6). The emergence of new strains of *Toxoplasma gondii* is related to the crossing of different strains during the sexual cycle in the intestines of infected felines (7).

P2X7 receptor (P2X7R) is a non-selective cationic ion channel, a member of the P2X receptors family. It comprises 595 amino acid residues, with an intracellular N-terminal region, an extracellular domain, two transmembrane helices, and a cytoplasmic C-terminal tail (8). The N-terminal region has a protein kinase C (PKC) phosphorylation site (9). During inflammation, the P2X7R is activated by extracellular ATP adenosine 3-phosphate(e-ATP) opening a nonselective cation channel. Millimolar concentrations of e-ATP can induce pore opening in cells (10). Activation of inflammation via ATP-P2X7R can promote the control of intracellular microorganisms such as Dengue virus, *Leishmania* sp., *Plasmodium* sp., *Pseudomonas aeruginosa*, *Porphyromonas gingivalis*, and *T. gondii* (11). The P2X7 receptor has significant genetic variability; these variations can promote loss of receptor function and increase susceptibility to some infections, such as toxoplasmosis (12–15).

The P2X7 receptor participates in different experimental models of *T. gondii* infection, *in vitro* with epithelial cells and macrophages and *in vivo* in systemic acute and chronic toxoplasmosis. In this context, infection with the *T. gondii* clonal types I and II showed that the activation of P2X7 receptor produced the inflammatory mediators as interleukin (IL)-1β, IL-12, IFN- γ, the reactive oxygen species (ROS), and lead to parasite control (16).

Several studies using susceptible mice with atypical Brazilian human *T. gondii* strain have been conducted in the context of reinfection (17–21). However, the atypical Brazilian strain infection process is still poorly understood (22, 23). In 1998, Dr. Flavia Cipriano Castro isolated a new strain of *T. gondii* in Brazil from the amniotic fluid of a pregnant woman in Minas Gerais named EGS (24). This strain presented an acute infection phenotype in BALB/c or C57BL/6 mice, with type I and III (virulent and low virulent, respectively) strain markers simultaneously. To obtain cysts, the animals must be treated with sulfadiazine, as described by Subauste and Hubal (25–27). It was shown to encyst in cell culture spontaneously (22, 25). Paredes-Santos (28) developed an EGS-DoubleCat (EGS-DC) strain with distinct stage markers. GFP fluorescence is detected during the bradyzoite phase, whereas mCherry fluorescence characterizes the tachyzoite stage. In the present work, we investigated the involvement of the P2X7 receptor during acute infection induced by the EGS atypical strain.

## 2 Material and methods

### 2.1 Animals

We used female 6–8-week-old C57BL/6 (WT) or P2X7 receptor knockout (P2X7^−/−^) mice from Jackson Laboratory (Bar Harbor, ME). Animals were housed in temperature-controlled rooms and received water and food *ad libitum*. The transgenic mice were kept in the Laboratory of Transgenic Animals of the Institute of Biophysics Carlos Chagas Filho in a particular room designed for these animals. The Commission for the Ethical Use of Research Animals (CEUA) from the Federal University of Rio de Janeiro approved all procedures (protocol number 086/19). The animals were euthanized using CO_2_ at a flow rate of 30% to 70% of the chamber volume per minute, following the guidelines recommended by the American Veterinary Medical Association (AVMA), and the protocol was approved by the CEUA (29).

### 2.2 Cyst maintenance, infections, and survival curves

Parasites were maintained in Carworth Farms 1 mice (CF1) mice by intragastric infection using a maximum volume of 100 μL phosphate-buffered saline (PBS) containing 20 brain cysts of *T. gondii* EGS or EGS-DC strain. After two days, the animals were treated with 0.5 mg/ml of sulfadiazine sodium salt (Sigma - S6387) in drinking water for ten days. Then, after 30 days, the animals were euthanized, and the brains were carefully removed and macerated in PBS. The cysts in brain homogenates were counted and used to infect other mice to keep the parasite reservoir or used for experiments. For experiments, the WT or P2X7^−/−^ mice were infected with three cysts by gavage. Infected mice were monitored daily for survival curve assay to address the infection susceptibility. The morbidity of infection was analyzed through the weight loss average by weighing the animals daily for eight days after infection. The group was separated into WT uninfected, WT infected, P2X7^-/-^ uninfected, or P2X7^-/-^ infected, each group with 6 mice.

### 2.3 Hepatic damage analysis

After euthanasia, the cardiac puncture was performed, and blood was collected using 0.1% EDTA-treated syringes. Blood samples were centrifuged at 700 × *g* for 10 min. Serum levels of free alanine aminotransferase (ALT) were evaluated using the Transaminase Alanine Aminotransferase Kinetics Kit, according to the manufacturer’s instructions (Bioclin, Rio de Janeiro, Brazil).

### 2.4 Histology and tissue measurements

Infected and noninfected mice were euthanized 8 days post-infection, and the length of the small intestine was measured. The spleen and liver were then weighed. Subsequently, the liver was rinsed with cold PBS. Organ samples were fixed in 40 g/L formaldehyde saline for at least 24 hours, dehydrated in ethanol and xylene (baths of 30 minutes each), and then embedded in paraffin. Paraffin sections (5 μm) were dewaxed and stained with hematoxylin and eosin (H&E). For the quantitative analysis of liver histology, seventeen high-quality images (2048 X1536 pixel buffer) were obtained with a digital camera (Evolution VR Cooled Color 13 bits, Media Cybernetics, USA) from histological sections stained with H&E (20x or 40x lens objective, for inflammation and hepatocyte injury, respectively). Liver damage and inflammation were assessed using a histological score adapted from Kleiner et al., 2005 and Gove et al., 2009 (30, 31). The hepatocyte damage index was assessed by evaluating the following parameters: steatosis (0–3), ballooning (0–2), vacuolization (0–3), and necrosis (0–2). The sum of the values attributed to each parameter was designated the liver damage index. To analyze the liver inflammation, the amount of portal inflammation (0–3), lobular inflammation (0–3), and the number of granulomas (0–3) were considered. The dimensions of the granulomas were evaluated under a 40x objective lens using ImageJ software.

### 2.5 Cytokine quantification from ileum explants

Samples of 1 cm of ileum were collected and washed with ice-cold sterile PBS. Then, the tissues were placed to the 96-well plate, before that added RPMI 1640 medium supplemented with 10% fetal bovine serum (Invitrogen-Gibco, Paisley, Scotland), 2 mmol/L l-glutamine, 50 mmol/L 2-mercaptoethanol, 10 mmol/L HEPES, 1000/L penicillin, and 100 mg/L streptomycin (all from Sigma Chemical Co., St. Louis, MO), and cultivated for 24 h, at 37°C in a 5% CO_2_ humidified incubator. Samples were centrifuged at 750 x *g* for 5 min, and for ELISA assays, 50 µl aliquots of supernatants were added to wells of 96-well plates (BD Biosciences − USA), and the levels of the inflammatory cytokines IL-1β, and IFN-γ were estimated using the cytokine dosage kit according to manufacturer’s protocol (BD Biosciences).

### 2.6 Cytokine assay from splenocyte culture

Spleen samples were dissociated using a 70-nm cell strainer (Discovery Labware, Franklin Lakes, NJ) in 1.5 mL ammonium chloride lysis buffer solution and were maintained for 7 min. The cells were washed with 40 ml ice-cold sterile PBS and were centrifuged twice at 2000 × *g* for 7 min. The cells were resuspended in RPMI 1640 medium supplemented as above and cultivated 1x10^6^ cells per well for 72 h at 37°C in a 5% CO_2_ humidified incubator. Samples were centrifuged at 750 x g for 5 min, and for ELISA assays, 50 µl aliquots of supernatants were added to wells of 96-well plates (BD Biosciences), and the levels of the inflammatory cytokines IL-1β, and IFN-γ were estimated using the cytokine dosage kit according to manufacturer’s protocol (BD Biosciences).

### 2.7 Parasite proliferation assay and parasite load

To evaluate the parasite proliferation, samples of 1 cm of ileum or the entire mice spleens were macerated with ice-cold sterile PBS and centrifugated at 250 x *g* for 5 min. The pellet was resuspended in 1 ml of RPMI 1640 medium supplemented as above, and 200 µl was added to monolayer LLC-MK2 culture, cultivated in 6-well plates at 37°C in a humid atmosphere with 5% CO_2_. After ten days, the cells were fixed and stained with fast panoptic (“Panotico Rapido kit,” LaborClin, Brazil). To determine the parasite load, the LLC-MK2 cell monolayer lysis analysis areas were quantified using ImageJ Fiji software (NIH).

The parasite load was also confirmed by real-time PCR (qPCR). The DNA was extracted from 1 cm of ileum macerated in lysis buffer (100 mM de Tris-HCl pH 8,5, 20 mM de NaCl, 5 mM EDTA, 0.2% de SDS), with 20 µg/mL proteinase K solution (Thermo Fisher Scientific Inc. – USA). The samples were placed in a water bath at 55°C overnight. The next day, the sample was incubated for 5 min to 95 °C and centrifugated at 2500 x *g* for 5 min. The supernatant was incubated in ice-cold absolute isopropanol and was added and mixed by inversion. Before the samples were placed in the freezer for 20 min, the samples were centrifuged at 7000 x *g* for 10 min. The pellet was incubated in 70% ice-cold ethanol and centrifuged at 7000 x *g* for 10 min. The samples were air-dried and resuspended in ultrapure water. The DNA from spleen and liver were extracted using TRIzol reagent (ThermoFisher Scientific Inc.). The qPCR was performed using a fluorescent probe HOT FIREPol^®^ EvaGreen^®^ qPCR Mix Plus (Promega - Brazil), 700 ng of DNA, and B1 *T. gondii* gene forward primer: GGAACTGCATCCGTTCATGAG, reverse primer: TCTTTAAAGCGTTCGTGGTC. The relative *T. gondii* DNA levels were quantified by interpolation with a standard curve obtained by multiple 10-fold serial dilutions with DNA extracted. The qPCR reaction for each gene was performed in triplicate. The PCR was performed using QuantStudio™ 3 System real-time PCR with the following cycling parameters: 95°C (10 min) followed by 40 cycles of 95°C (30 s) and 60°C (1 min).

### 2.8 *In vivo* fluorescence imaging

The animals infected with 10 cysts, the EGS-DC strain, after 8 days, were analyzed by detecting the fluorescence in the whole animal using the IVIS Lumina image system (Revvity). All mice were immediately anesthetized in an oxygen-rich induction chamber with 2% isofluorane, and images were captured through the Cy5.5 emission filter, filter position 4, and 640 nm excitation filter. The fluorescence images were obtained with mice in the ventral position and continued anesthesia during the entire imaging process using a nose cone isofluorane-oxygen delivery device in the light-tight chamber. To acquire the fluorescence images, some parameters were considered based on the level of fluorescence emission, such as the automatic time of exposure, which ranged from a few seconds to 5 min, binning = 4, f/stop =2, and the field of view (FOV)= 12.5 cm, with automatic focus. The Living Image software 3.2 (Revvity) automatically co-registered the fluorescent images, which were taken in darkness and displayed in pseudo-colors that represent the intensity of the signal refund the photographic image, generating an overlay image. Fluorescence was expressed as the total or average radiant efficiency ([photons/s/cm2/steradian] = [p/sec/cm²/sr]) of selected regions of interest (ROIs).

### 2.9 Expression of immune mediators

According to the manufacturer’s instructions, 1-cm ileum samples were macerated using TRIzol reagent (ThermoFisher Scientific Inc.), and the cDNA was constructed using the High-capacity kit (ThermoFisher Scientific Inc.). The real-time PCR (qRT-PCR) was performed using HOT FIREPol^®^ EvaGreen^®^ qPCR Mix Plus (Promega) to analyze the following targets: NLRP3, IL-1β, TNF, IFN-γ, Foxp3, and β-actin (**Supplementary Methods 1**). The mRNA levels were normalized to the expression levels of the control genes β-actin. The ΔΔCt method was used for the data analysis to determine the fold change of all target genes in each sample with 95% confidence. The qRT-PCR reaction for each gene was performed in duplicate in each experiment. The PCR was performed using QuantStudio™ 1 System real-time PCR with the following cycling parameters: 95°C (10 min) followed by 40 cycles of 95°C (30 s) and 60°C (1 min).

### 2.10 Immunophenotyping of the small intestine and spleen cells

The lamina propria and splenocytes were collected according to **Supplementary Methods 2** and **3**. Cells underwent two washes in PBS containing 0.5% BSA, 1 mM EDTA, and 2 mM sodium azide. Subsequently, cells (3 × 10^5^) were stained using standard procedures. The employed antibodies were: CD45-PE-Cy7, CD3-FITC, CD4-PE, and Foxp3-APC. Cell samples were analyzed on a BD Fortessa Analyzer cytometer. FlowJo™ software was utilized for analyses by gating the lymphocyte population based on relative size and granularity. Results were presented as the percentage and absolute numbers of each lymphocyte population. Gating definitions were established using fluorescence minus one controls, where cells are stained with all antibodies, excluding the one of interest for gating.

### 2.11 Statistical analysis

A two-tailed t-test was used to compare two groups, whereas multiple comparisons were performed by one-way analysis of variance followed by the Tukey post-test. Tukey’s multiple comparisons test compared two or more groups using ordinary one-way ANOVA. All statistical analyses were performed using GraphPad Prism 5 (GraphPad, La Jolla, CA).

## 3 Results

### 3.1 The P2X7 receptor contributes to the parasite control in the intestine of EGS-infected mice

**Figure 1:**
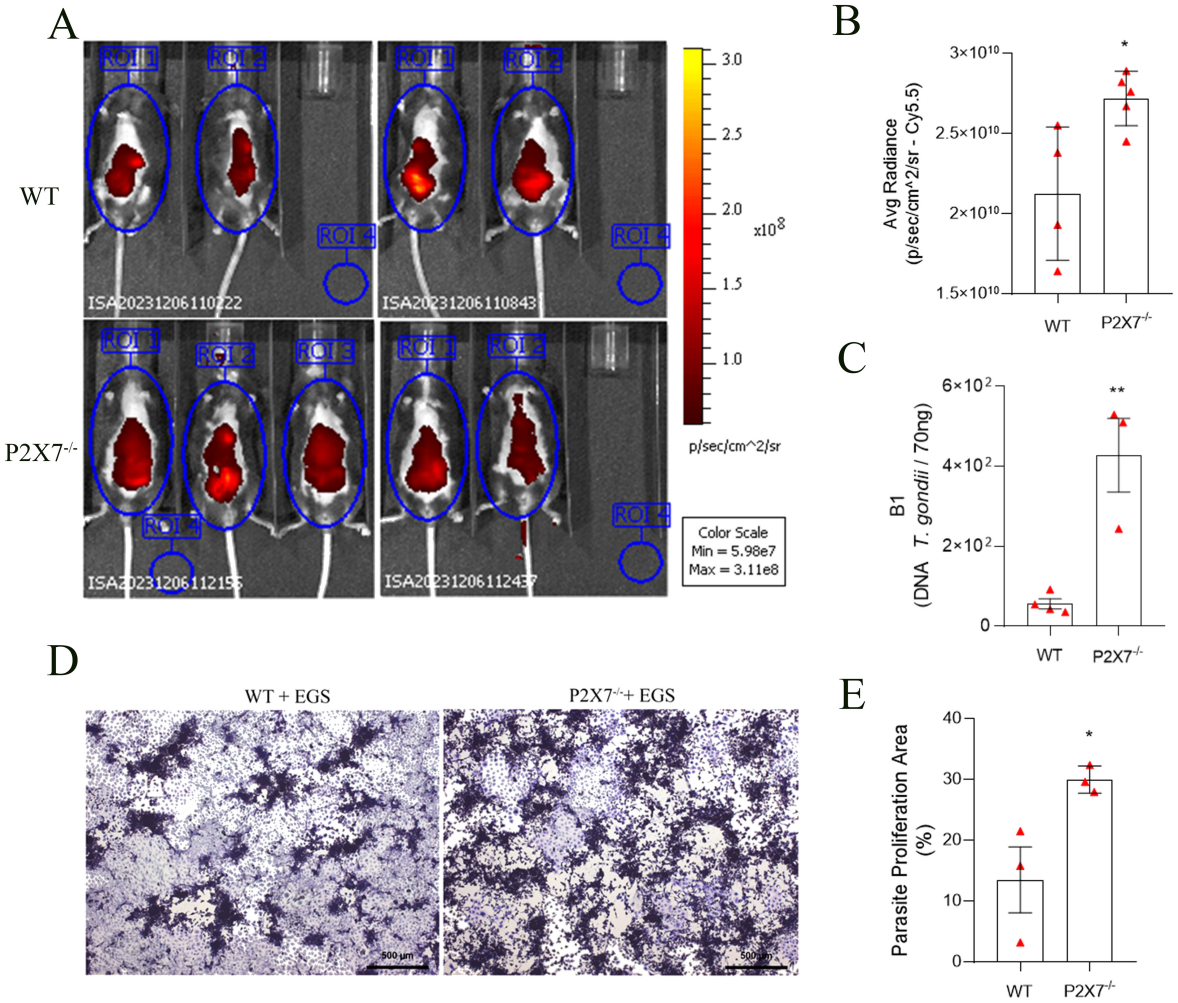
The P2X7 receptor controls the parasite load in C57BL/6 mice. **(A)** Fluorescence images were acquired using four WT-infected mice and five P2X7^-/–^infected mice. **(B)** averaged radiance in equal-sized regions of interest (ROI). Mice were placed inside the IVIS lumina chamber and imaged in the ventral, and the fluorescence was measured in the ROI. **(C)** The B1 gene expression was analyzed by qPCR. **(D)** Representative photomicrograph of a plaque assay with LLC-MK2 cells. **(E)** Quantification of proliferation parasite area. **(B)** Data represent the mean and SD of four WT mice and five P2X7^-/-^ mice analyzed by unpaired t-test. **(C)** Data represent the mean and SD of four WT mice and three P2X7^-/-^ mice analyzed by unpaired t-test. **(D)** Data represent the mean and SEM of forty-three photos from three animals analyzed by unpaired t-test. (*) Significance compared to the WT group. (*) p < 0.05; (**) p < 0.01.

On the eighth day of infection with the EGS-DC *T. gondii* strain, the presence of tachyzoites in the ventral region of mice was evaluated using the Cy5.5 filter in IVIS. The P2X7 knockout (P2X7^-/-^) mice exhibited a higher fluorescence rate in the ventral region than the wild-type (WT) cohort (**Figure 1A**). Quantification of the ROI confirms greater fluorescence in P2X7^-/–^infected with the EGS strain (**Figure 1B**). This finding suggests that P2X7^-/-^ animals are more susceptible to parasitic proliferation.

On the ninth day post-infection, the P2X7-/- mice group began to exhibit mortality. However, by the eleventh day, both cohorts died (**Supplementary Figure 1A**). Additionally, all infected cohorts displayed weight loss compared to the uninfected control, with no significant alteration noted among the infected groups (**Supplementary Figure 1B**). Considering that inflammatory bowel diseases conventionally result in diminished intestinal length, this metric served as an indicator of intestinal inflammation (32–34). Significant reductions in small intestine lengths were evident in both infected animal cohorts (**Supplementary Figure 1C**). These results indicated that the inflammatory profile induced by EGS strain infection is present in all studied cohorts.

Analyzing the parasite load by qPCR through the detection of the B1 gene, an increase in parasite load in the intestines of P2X7^-/-^ infected mice was observed compared with the WT-infected mice (**Figure 1C**). Subsequently, a parasite proliferation assay was conducted using parasites obtained from the small intestine added to LLC-MK2 cell monolayers (**Figure 1D**). Quantification of the parasite proliferation area confirmed this difference and showed that the intestines of P2X7^-/-^ infected mice had a higher parasite burden that WT-infected intestines (**Figure 1E**). These findings demonstrate the virulence of the EGS strain and its potential to induce morbidity in infected animals while inciting intestinal inflammation.

### 3.2 The EGS strain infection modifies inflammatory parameters and subpopulations of inflammatory cells in the mouse small intestine

**Figure 2:**
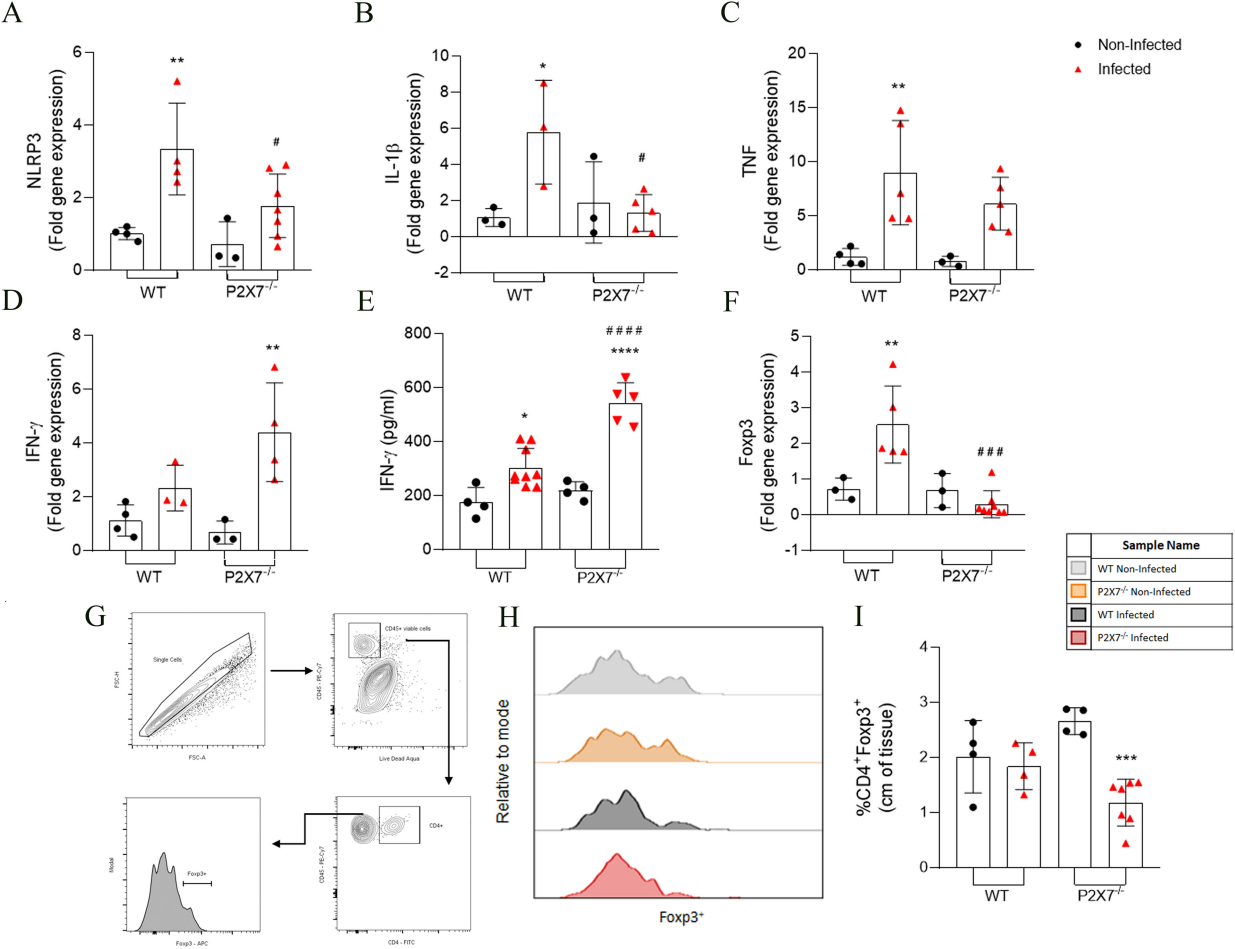
The EGS strain infection changes the inflammatory parameters and subpopulation cells in the intestine of infected animals. **(A–D, F)** RT-qPCR analyzed the NLRP3, IL-1β, TNF-α, IFN-γ, and Foxp3 gene expression. **(E)** Secretion levels of IFN-γ were analyzed in the supernatant in the ileum explant. **(G)** The gating strategy of T reg population (CD4^+^Foxp3^+^) cells analysis in lamina propria. **(H)** The histogram showed the CD4^+^Foxp3^+^ cell. **(I)** Quantification of CD4^+^Foxp3^+^ cells. **(A–F)** Data represent the mean and SD of 3 - 6 animals. Analyzed by ordinary one-way ANOVA, Tukey’s multiple comparisons test. **(I)** Data represent the mean and SD of 4 WT, WT-infected, and P2X7^-/-^ mice and 7 P2X7^-/–^infected mice. Analyzed by ordinary one-way ANOVA, Tukey’s multiple comparisons test. (*) Significance compared to the non-infected group; (#) significance between infected groups. (*/#) p < 0.05; (**) p < 0.01; (***) p < 0.001; (###) p < 0.001; (****/####) p < 0.0001.

Analyses of cytokines gene expression in the ileum showed that EGS infection promoted increased expression of NLRP3, IL-1β, and TNF in WT-infected when compared with WT-uninfected mice (**Figures 2A–C**). However, P2X7^-/–^infected mice showed fewer NLRP3 and TNF than the WT-infected mice (**Figures 2A, B**). The TNF in P2X7-infected mice did not exhibit significant alterations (**Figure 2C**).

The IFN-γ gene expression was analyzed. An increase was observed in P2X7^-/–^infected mice, whereas WT-infected mice did not exhibit significant alterations (**Figure 2D**). Regarding the inflammatory cytokine secretion, an increase of IFN- γ in the intestine was observed in all infected groups compared to the non-infected, being even higher in P2X7^-/–^infected mice (**Figure 2E**). The levels of IL-1β secretion did not show a significant difference between WT-infected and P2X7^-/–^infected mice (data not shown).

Examining a T regulatory transcription factor (Foxp3) revealed that Foxp3 expression increased in WT-infected mice, whereas in P2X7^-/–^infected mice, a reduction was observed compared to WT-infected mice (**Figure 2F**). Immunophenotyping of the ileum lamina propria was analyzed using a gating strategy as illustrated in **Figure 2G**. This result showed a reduction in the frequency of the CD4^+^Foxp3^+^ cells only in P2X7^-/-^ mice, but WT-infected mice exhibited no significant alterations (**Figures 2H, I**). These data indicate that infection with the EGS strain also causes an increase in the inflammatory parameters; however, in the absence of the P2X7 receptor, there is a dysregulation of the inflammatory mediators, possibly associated with the increased parasitic load and tissue damage previously observed in **Figure 1**.

### 3.3 The EGS strain induces damage and inflammation in the liver during acute infection

**Figure 3:**
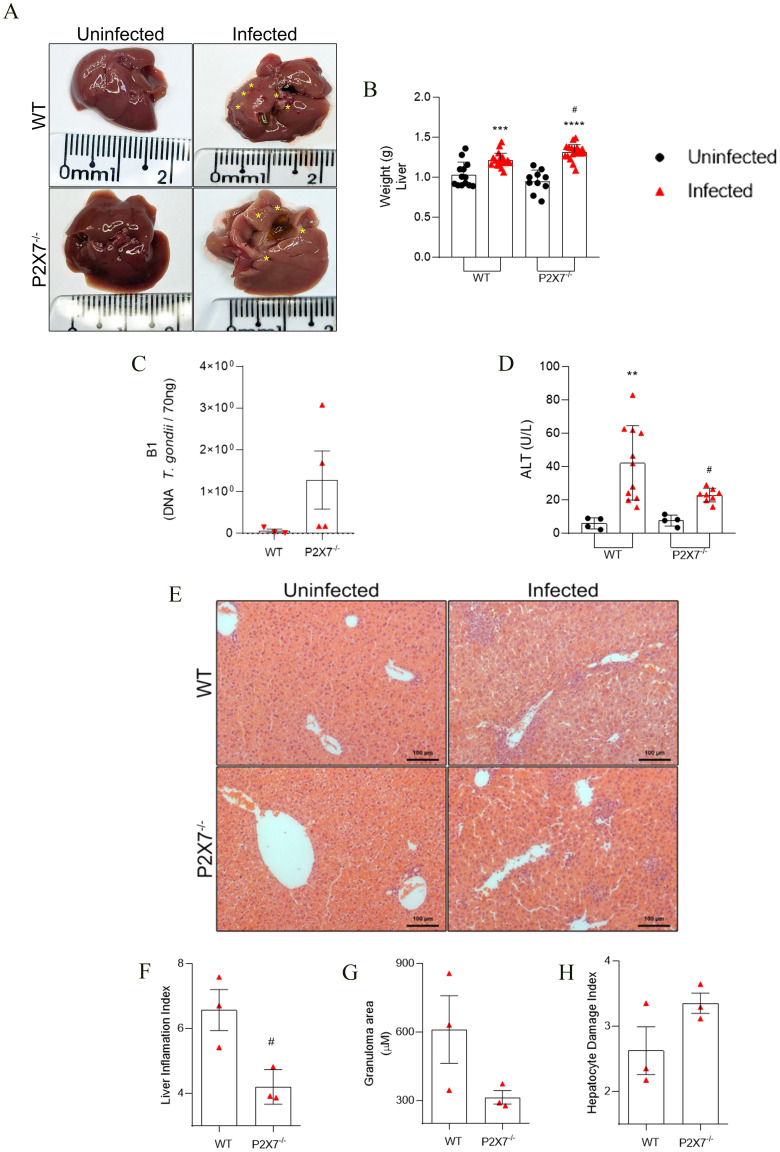
The EGS strain promotes liver damage and liver inflammatory infiltration. **(A)** Liver photomicrography, the asterisks show points of necrosis in the tissue. **(B)** liver weight weighed. **(C)** The B1 gene expression by qPCR. **(D)** Blood levels of ALT for systemic and liver tissue damage. **(E)** Photomicrography of liver tissue stained with hematoxylin and eosin. **(F)** Graphic showing liver inflammation index quantification. **(G)** Graphic showing granuloma area mensure. **(B)** Data represent the mean and SD of 13 WT and 10 P2X7^-/-^ uninfected mice, and 19 WT and 20 P2X7^-/–^infected mice in three different experiments, analyzed by ordinary one-way ANOVA, Tukey’s multiple comparisons test. **(C)** Data represent mean and SD of three or four animals analyzed by unpaired t-test. **(D)** Data represent the mean and SD of 4 uninfected mice, 11 WT-infected mice, and 8 P2X7^-/–^infected mice in two different experiments, analyzed by ordinary one-way ANOVA, Tukey’s multiple comparisons test. **(F-H)** Data represent the mean and SEM of seventeen images from three animals analyzed by unpaired t-test. (*) Significance compared to the non-infected group; (#) significance compared between infected groups. (#) p < 0.05; (**) p < 0.01; (***) p < 0.001; (****/) p < 0.0001.

Macroscopic analysis of the liver revealed necrotic zones in infected mice (**Figure 3A**), which were also detected histologically. An increase in organ weight was noted in the infected mice, with a more pronounced effect observed in the P2X7^-/–^infected group compared to the WT group (**Figure 3B**). The parasitic load in the liver, assessed through real-time PCR, was higher in the P2X7^-/–^infected compared to the WT-infected animals (**Figure 3C**). Evaluation of tissue damage showed that blood levels of alanine transferase (ALT) were elevated in the infected WT mice compared to the P2X7^-/-^, however, there was no significant difference between P2X7^-/–^infected group from the control uninfected (**Figure 3D**). Histological analysis of the livers (**Figure 3E**) shows that the inflammation index is lower in P2X7^-/–^infected group compared to WT-infected (**Figure 3F**). Additionally, there is a trend toward a reduction in granuloma size in P2X7^-/–^infected (**Figure 3G**). However, in the hepatocyte damage index, there was no significant difference (**Figure 3H**). These findings highlight the significant role played by the P2X7 receptor during infection with the EGS strain, primarily through the recruitment of immune cells that contribute to controlling parasitic proliferation.

### 3.4 P2X7 receptor-mediated control of parasite proliferation in the spleen of infected animals

**Figure 4:**
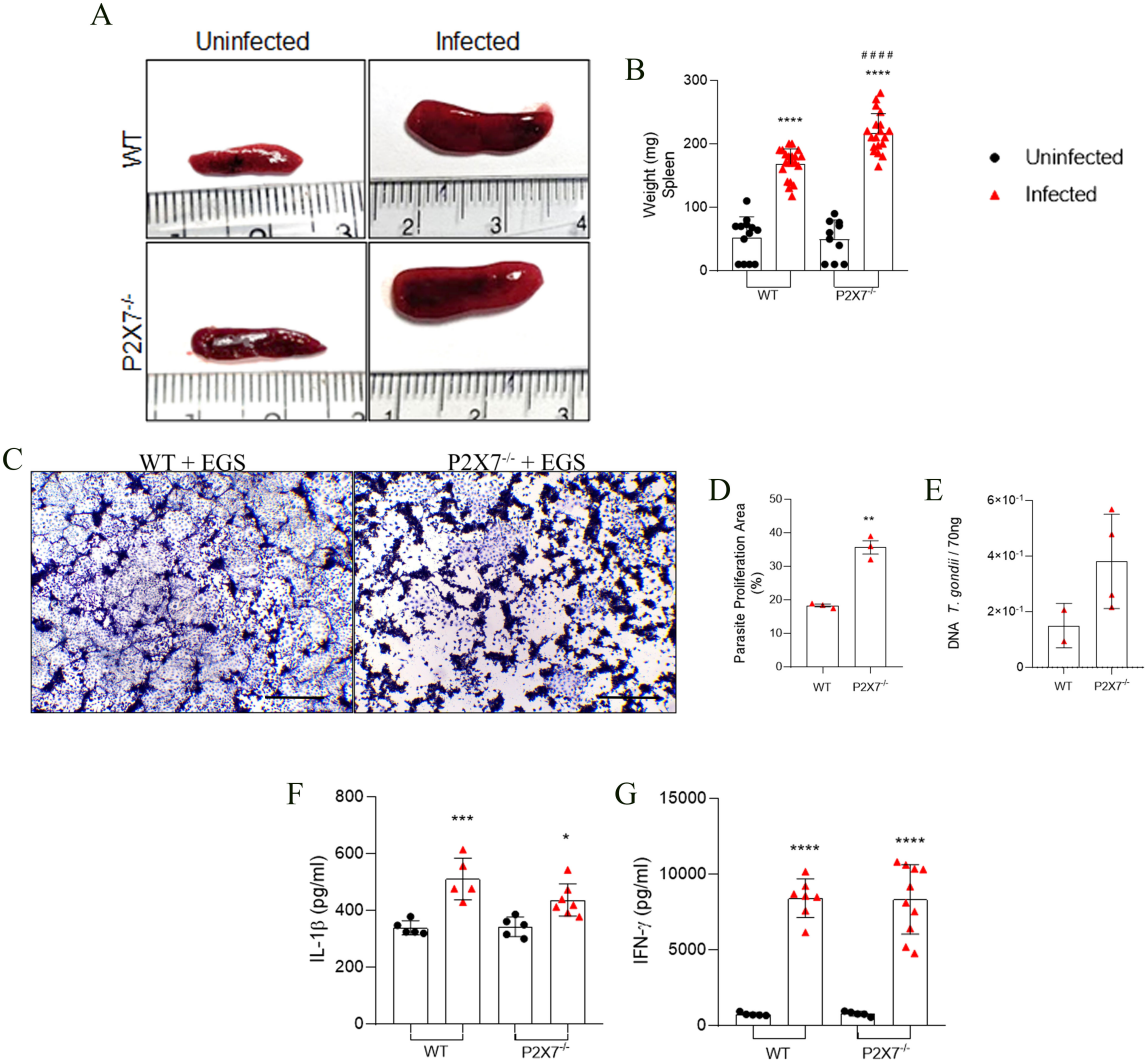
The P2X7 receptor controls the *T. gondii* parasite proliferation in mice spleen. **(A)** Spleen photomicrography. **(B)** Graphic of spleen weight. **(C)** Representative photomicrograph of a plaque assay with LLC-MK2 cells. Scale bar 50 µm. **(D)** Quantification of proliferation parasite area. **(E)** Parasite load quantification using the B1 gene expression analyzed by qPCR. **(F, G)** Levels of IL-1β, and IFN-γ cytokines in the supernatant in splenocytes culture after 72 (h). **(B)** Data represent the mean and SD of 13 WT and 10 P2X7^-/-^ uninfected mice, and 19 WT and 20 P2X7^-/–^infected mice in three different experiments, analyzed by ordinary one-way ANOVA, Tukey’s multiple comparisons test. **(D)** Data represent the mean, and SEM of forty-three photos from three different animals analyzed by unpaired t-test. **(E)** Data represent the mean and SD of 3 - 6 animals, analyzed by ordinary one-way ANOVA, Tukey’s multiple comparisons tests. **(F, G)** Data represent the mean and SD of 3 - 6 animals, analyzed by ordinary one-way ANOVA, Tukey’s multiple comparisons test. (*) Significance compared to the non-infected group. (**) p < 0.01; (****) p < 0.0001.

Examination of spleen infection in the mice revealed that EGS infection induces enlargement of spleen size in both WT and P2X7^-/-^ infected mice (**Figure 4A**). An increase in spleen weight was observed following infection in WT animals, with a greater magnitude observed in the infected P2X7^-/-^ group (**Figure 4B**). T-cell assessment in the spleen was conducted using flow cytometry, with the gating strategy shown (**Supplementary Figure 2B**). A decrease in lymphocytes (CD45^+^CD3^+^) (**Supplementary Figures 2C–E**) and CD4^+^ T cells (CD45^+^CD3^+^CD4^+^) (**Supplementary Figures 2F–H**) in both percentage and absolute number was observed in all infected groups.

LLC-MK2 cells were exposed to the macerate of spleens from WT-infected mice, showing small areas of parasitic proliferation when compared to cells exposed to the macerate from spleens of P2X7^-/-^ mice (**Figure 4C**). The parasite proliferation figure demonstrates this significant difference (**Figure 4D**). To confirm this observation, real-time PCR was conducted for parasite load analysis; there was a trend to higher parasitic burden in the spleen of P2X7^-/-^ mice in comparison with WT (**Figure 4E**). Regarding cytokine production, splenocytes from infected animals exhibited the capability to secrete IL-1β and IFN-γ in the supernatant of spleen explants after 24 hours in WT and P2X7^-/-^ infected mice (**Figures 4F, G**, respectively). These findings suggest that the parasite can modulate the quantity of T cells in the spleen independently of the P2X7 receptor and induce cytokine production. Nevertheless, the P2X7 receptor remains crucial for controlling parasitic proliferation in the spleen.

## 4 Discussion

*Toxoplasma gondii* is a unicellular pathogen parasite spread across all continents. The clinical outcome of toxoplasmosis is associated with the immunological status of host and parasite strains. A systematic review showed that from 1995 to 2017, most human toxoplasmosis cases were related to the Type II strain in North America and Europe (less virulent and cytogenetic) (35). South America has many atypical isolates that can cause unexpected symptoms for this infection in immunocompetent patients, with high rates of ocular manifestation and greater severity in congenital toxoplasmosis (35). In Chile, 11 of 15 symptomatic children presented ocular manifestations. In Colombia, of 25 children studied, 57% had retinochoroiditis, and 64% of cases had neuroradiological changes (36). Brazil harbors atypical *T. gondii* strains associated with severe ocular and systemic complications in immunocompetent and congenitally infected patients (3). In Southern Brazil, some municipalities record 74.5% of pregnant patients seropositive for *T. gondii* (37). However, infections with atypical Brazilian strains were less explored. Therefore, this study represents the starting point for understanding purinergic signaling in acute infections caused by atypical Brazilian strain.

The mucosal immune response against the *T. gondii* type I strain involves the P2X7R/NLRP3 pathway, producing IL-1β secretion and inhibiting parasite proliferation (38). Our findings showed that IL-1β transcription was reduced in the small intestine in P2X7^-/-^ animals. However, in the spleen, the secretion of IL-1β was similar to WT-infected mice. This indicates a difference in infection dynamics between the EGS and Type I strains, which required further investigation. The literature showed that atypical strains found in Colombia decreased the IFN-γ expression in the aqueous humor of patients with severe ocular toxoplasmosis, suggesting that atypical strains could inhibit the expression of specific Th1-type cytokines, thereby increasing the severity of toxoplasmosis (39). Concerning the intestine, several *T. gondii* strains induce ileitis, characterized by inflammation, particularly in the terminal portion of the small intestine—the preferred parasite entry site in the host (40, 41). Our results demonstrated that the production of IFN- γ in the intestine and spleen of P2X7^-/-^ mice infected with the EGS strain could not control parasite growth. However, protein quantification will provide a more robust cause-and-effect correlation regarding cytokine production in the intestines of mice infected with the EGS strain.

Other *in vitro* mechanisms activated through the ATP-P2X7R axis during *T. gondii* infection are lysosomal fusion, activation of the inflammasome NLRP3, and secretion of cytokines as IL-1β, TNF, IL-6, and CCL5, that promote the control of parasite proliferation (42, 43). In the acute phase of infection with a type I strain, the absence of the P2X7 receptor in mice reduces pro-inflammatory cytokines in the peritoneal cavity washes. These cytokines are essential to control the parasite load in the peritoneal cavity and prevent damage to the spleen and liver (44). In the intestines of the infected mice, the protection against *T. gondii* infection requires the P2X7-NLRP3 axis, protection of intestine inflammation, and systemic immune responses (41). Our work corroborates the role of the P2X7 receptor in activating the axis of immunity during infection, as occurs in several typical strains of *T. gondii*. This receptor controls the expression of inflammatory markers, such as NLRP3 and IL-1β, which helped control parasitic replication during infection with the EGS strain. *T. gondii* displays an array of effector proteins involved in the immune clearance (45). *T. gondii* secretes important molecules that can downregulate Foxp3^+^ expression during pregnancy (46). These dysregulations in the lymphocyte can affect the production of inflammatory cytokines involved in controlling infection. Our results demonstrate that the EGS strain can reduce FOXP3^+^ cells in the intestine-infected cells, independent of the P2X7 receptor.

The literature indicates that the P2X7 receptor is upregulated in various liver cells during non-alcoholic steatohepatitis, contributing to hepatocyte apoptosis, inflammation, and fibrosis (47). In sepsis, deleting the P2X7 receptor attenuates tissue damage by producing free radicals and inflammatory cytokines in the liver (48). Analyzing Egyptian patients with chronic liver disease, it was observed that 30% of patients infected with *T. gondii* showed a significant increase in liver enzymes compared with noninfected patients (49). A systematic review of pooled prevalence rates of *T. gondii* in patients with liver diseases (35.97%) confirmed the positive connection between *T. gondii* infection and chronic liver diseases (50). In this study, *T. gondii* infection increased liver size, and the liver inflammation index was observed in WT-infected mice. The absence of the P2X7 receptor was crucial for recruiting immune cells to the liver, as shown by the liver inflammation index and granuloma size. Therefore, the liver inflammation index is decreased in P2X7^-/–^infected mice. The increase in parasite load may be associated with higher levels of steatosis and necrosis in the livers of infected animals, parameters used to assess the liver damage index.

In the infection induced by the ME-49 and RH strains, the presence of the P2X7 receptor has proven significant for controlling tissue damage in both models of animals infected intraperitoneally or by gavage (25, 41, 51). The intraperitoneal infection with the RH strain induced an augmentation in the organs of the infected animals, such as the liver and spleen, along with an elevated parasitic load in the liver of P2X7^-/-^ animals, indicating greater dissemination of the parasite within the host (44). Our findings suggest that P2X7 receptor in the liver may play a role in modulating the inflammatory response and controlling parasite burden. Further investigation is warranted to fully understand the mechanisms involved and their implications for therapeutic strategies.

Concerning the analysis of the spleen, our data indicated that in C57BL/6 mice infection, the EGS strain reduced splenic lymphocytes, and in P2X7^-/–^infected mice, the parasitic load was not controlled. Previous studies with BALB/c mice showed that tachyzoites of the RH strain could infect the spleen, leading to apoptosis even at very low infection levels (52, 53). Other studies showed that the ME-49 strain infection caused severe homeostatic disorders in the spleen, such as diffuse inflammation in the white and red pulp and atrophy of the white pulp (54). These studies suggest apoptosis may be the mechanism behind the reduction of lymphocytes in the spleen in the context of EGS infection. However, further investigations are necessary to elucidate these data better.

In conclusion, our results demonstrated that EGS strain infection in the mice caused an immune modulation independent of the P2X7 receptor with acute inflammation and cytokine disorder production. However, the presence of the P2X7 receptor promoted protection against parasitic growth in various organs of the infected animals. This study serves as a starting point in understanding the immunopathology of infection by a Brazilian strain of *T. gondii* in the context of purinergic signaling, highlighting the severity of infection caused by atypical strains.

## Data Availability

The raw data supporting the conclusions of this article will be made available by the authors, without undue reservation.
