# Supplementary material for: Inflammatory response and parasite regulation in acute toxoplasmosis: the role of P2X7 receptor in controlling virulent atypical genotype strain of Toxoplasma gondii
Source: Front Immunol. 2024 Aug 29;15:1452828. doi: 10.3389/fimmu.2024.1452828 (PMC11390460; doi:10.3389/fimmu.2024.1452828)
Supplement: Supplementary file 1 [file DataSheet1.docx]

Supplementary Material

# Supplementary Methods 1: Primer List

| *NLRP3* | 5’- GCTGCTCAGCTCGACCTCT-3’;  5’-AGGTGAGGCTGCAGTTGTCT-3’ |
| --- | --- |
| *IL-1β* | 5’-TTCAGGCAGGCAGTATCACTC-3’;  5’-CCACGGGAAAGACACAGGTAG-3’ |
| *TNF-α* | 5’- GGT CCC CAA AGG GAT GAG AAG TTC-3’;  5’-CCA CTT GGT GGT TTG CTA CGA CG-3’ |
| *IFN-γ* | 5'-ACAGCAAGGCGAAAAAGGATG-3';  5'-TGGTGGACCACTCGGATGA-3' |
| *Foxp3* | 5’- AGCAGTGTGGACCGTAGATGA-3’;  5’- GGCAGGGATTGGAGCACTT-3’ |
| *β-actin* | 5’-TATGCCAACACAGTGCTGTCTGG-3;  5’-TACTCCTGCTTGCTGATCCACAT-3’ |

# Supplementary Methods 2: Lamina Propria Isolation

Collected a 5 cm segment of the small intestine and washed it with buffer A (5% fetal bovine serum, 1 M HEPES, and 2 L of Hanks), followed by cutting it into small pieces. The tissue pieces were placed in a tube and vortexed with 10 mL of buffer A. After removing the supernatant, buffer B (25 mL HEPES, 0.5 M EDTA, and 1 L of Hanks) was added. The pellet was resuspended in 10 mL of buffer C (10% fetal bovine serum, 1 M HEPES, 0.5 M EDTA, 1 mM DL-dithiothreitol (DTT-Sigma), and 500 mL Hanks) preheated to 37ºC, and incubated at 37ºC for 15 min, with vortexing every 5 min. The supernatant was removed by passing it through a cell strainer, and the tissue was placed in a tube with 10 mL of buffer A.

The tissue was then placed on a sieve and patted dry with paper towels to remove the buffer. Subsequently, the tissue was cut into very small pieces. These pieces were transferred to a new tube with 10 mL of buffer A, centrifuged for 7 min at 600 x g at 4 ºC, and the supernatant was discarded. A digestion solution (Collagenase: 167 µg/mL; DNAse: 30 µg/mL; in RPMI medium) was added and incubated at 37ºC for 1 hour on a shaker with vortexing every 15 min. After centrifugation for 7 min at 600 xg at 4ºC, the supernatant was discarded, and the pellet was resuspended in 10 mL of RPMI medium with 10% fetal bovine serum.

Further centrifugation and resuspension steps were performed, first in cold buffer A, and then the pieces were filtered through a 100 µm cell strainer. After centrifugation and removal of the supernatant, the pellet was resuspended in 10 mL of buffer B. This filtration step was repeated with a 40 µm cell strainer. The final steps involved centrifugation, removal of the supernatant, and resuspension in 1 mL of FACS buffer (PBS + 0.5% BSA + 1 mM EDTA + 2 mM sodium azide).

# Supplementary Methods 3: Splenocytes Isolation

The spleens were extracted and kept in PBS on ice until tissue dissociation. Using a 40 µm cell strainer placed on a 90 mm petri dish, 1 mL of Ammonium-Chloride-Potassium (ACK) Lysing Buffer (Thermofisher-Brazil) was added, and the tissue was macerated. The volume was transferred to a tube and incubated for 7 minutes for red blood cell lysis at room temperature. After lysing, the sample was washed twice with sterile PBS, centrifuged at 900 x g for 7 minutes, and then resuspended in FACS buffer.

# Supplementary Figures and Tables

##
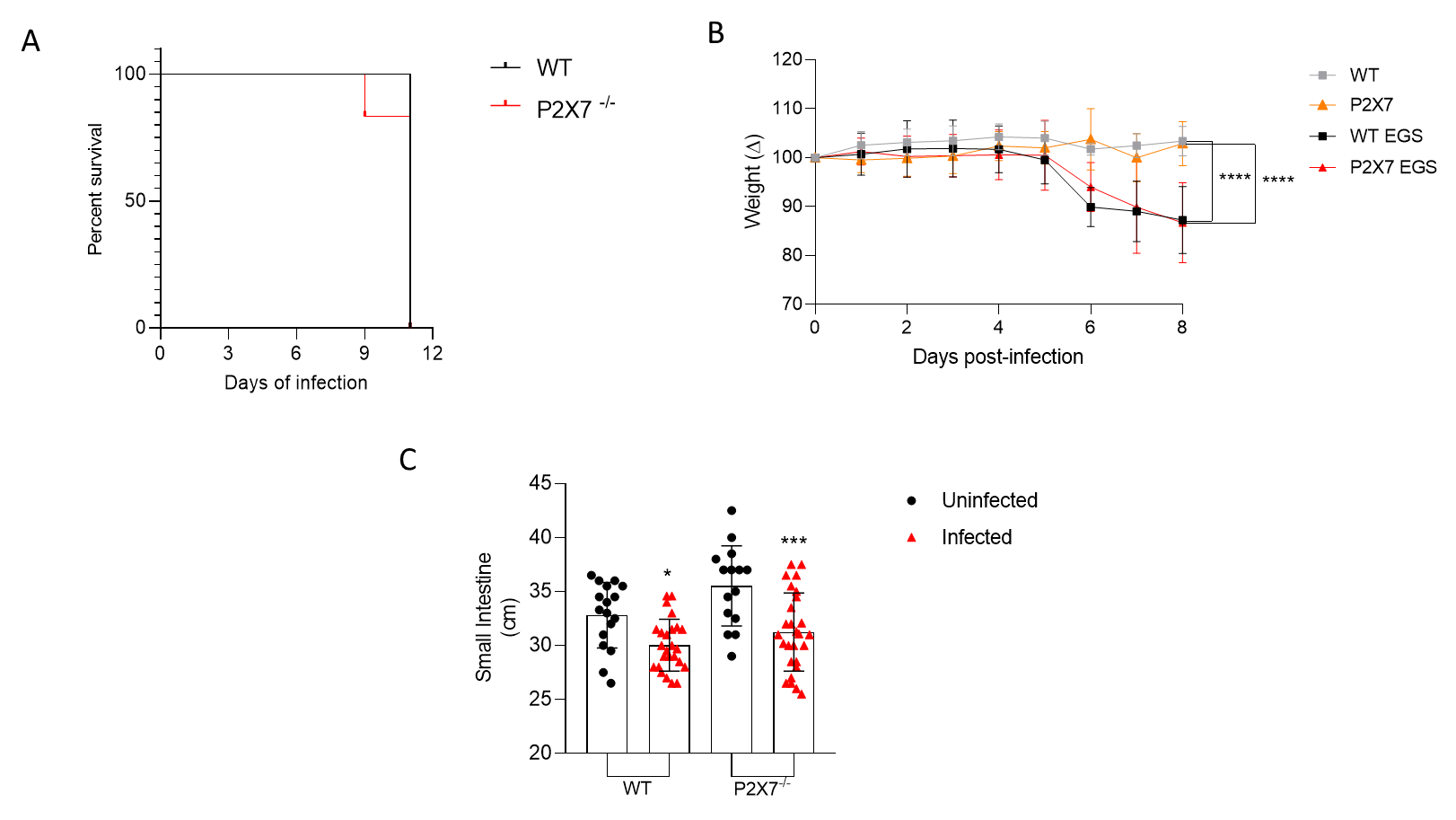
Supplementary Figure 1

**Supplementary Figure 1.** EGS strain promotes mortality and weight loss. The mice were infected via gavage with 3 cysts of the *T. gondii* – EGS strain. (A) Mortality was monitored for 11 days. We chose the 8^th^ day of euthanasia. (B) The weight of animals reduces after 6 days of infection, significantly less than the uninfected group after 8 days. (C) After 8 days of infection, we found a decreased tissue length indicative of inflammation. (A) Data represent the mean and SD of 6 animals per group comparison of survival curve with Long-rank (mantel-cox) test. (B - C) Data represent the mean and SD of 13 animals in the WT group; 23 animals in the WT EGS group; 10 animals in P2X7^-/-^ group; and 20 animals in P2X7^-/-^ EGS group. (B) Analyzed by 2-way ANOVA with Tukey’s multiple comparisons test. (C) Analyzed by ordinary one-way ANOVA, Tukey’s multiple comparisons test. (*) Significance compared with the uninfected group. (*) p<0.05; (****) p <0.0001.

## Supplementary Figures 2


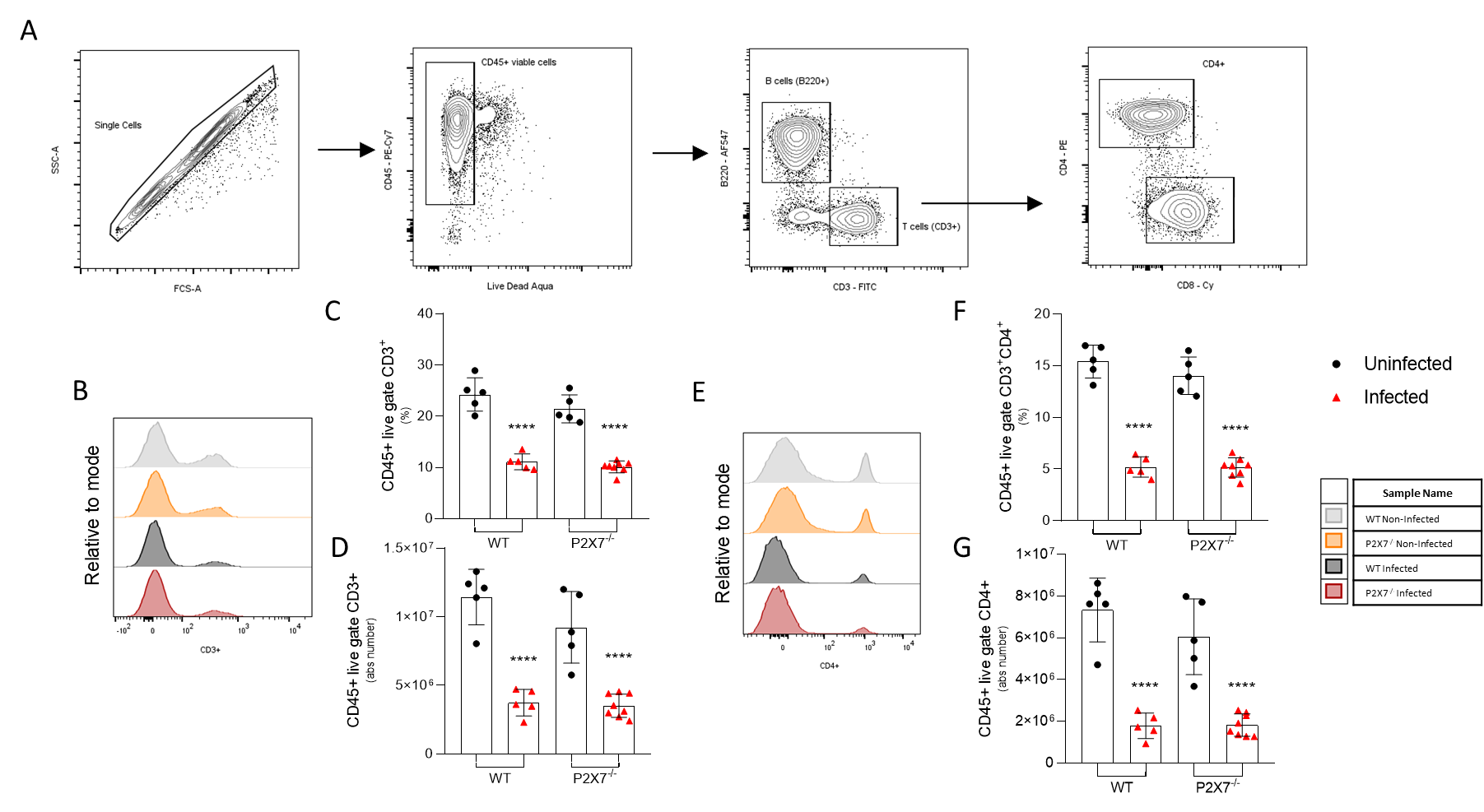


**Supplementary Figure 2.** EGS strain changes in the red pulp and cellularity of the spleen (A) Gating strategy of CD45^+^CD3^+^ and CD45^+^CD3^+^DC4^+^ cells analysis. (B) The histogram showed CD45^+^CD3^+^ cells. (C) The quantification graphic showed the percentual (%) of CD45^+^CD3^+^. (D) The quantification graphic showed the absolute number (abs number) of CD45^+^CD3^+^. (E) The histogram showed CD45^+^CD3^+^CD4^+^ cells. (F) The quantification graphic showed the percentual (%) of CD45^+^CD3^+^CD4^+^ cells. (G) The quantification graphic showed the absolute number (abs number) of CD45^+^CD3^+^CD4^+^ cells. Data represent the mean and SD of 5 WT, WT-infected, and P2X7^-/-^ mice and 8 P2X7^-/-^-infected mice. Analyzed by ordinary one-way ANOVA, Tukey’s multiple comparisons test. (*) Significance compared to the uninfected group. (****) p < 0.0001.
